# Transmigration of an Impacted Mandibular Canine: A Report of Two Cases

**DOI:** 10.7759/cureus.74444

**Published:** 2024-11-25

**Authors:** Veeresh Matmari, Himasagar Ellampalli, Jhansi Rani Lotavath, Avanthika Vinod

**Affiliations:** 1 Dentistry, Employees State Insurance Corporation (ESIC) Medical College and Hospital, Chennai, IND

**Keywords:** impaction, orthopantogram, surgical extraction, transmigration, transplantation

## Abstract

Transmigration of an impacted mandibular canine is a rare entity. Published cases are scarce. The etiology and pathogenesis remain unclear. The most opted treatment is surgical extraction of the affected tooth. In this case report, we described two cases of a transmigrated and impacted mandibular canine, treated by surgical extraction. The incidence, etiology, clinical findings, investigations, and treatment options are discussed here.

## Introduction

Impaction is defined as the failed eruption of a permanent tooth with a completely developed root, while transmigration is defined as the migration of an impacted tooth across the midline [1]. Ando et al. were the first to use the term "transmigration" [2]. Camilleri et al. said that the mandibular permanent canine is the only tooth in the dental arch reported to migrate across the midline [3].

Umashree et al. observed that the incidence of transmigration has increased over the past 50 years with the introduction of dental panoramic tomography [4]. Aydin et al. reported a panoramic radiographic survey of 4500 patients in a Turkish subpopulation, which revealed 14 cases of canine transmigration, out of which six were maxillary and eight mandibular canines, with an incidence of 0.31% [5]. Dalessandri et al., in their review, reported that the incidence of mandibular canine impaction ranges from 0.92-5.1 percent, while that of canine transmigration ranges from 0.1-0.31 percent [1]. Javid reported a radiographic survey of 1000 students, which revealed only one case of transmigrated impacted mandibular canine [6]. Sathyanarayana et al., in their systematic review, found the prevalence of mandibular canine impaction to be 0.008-1.29 percent, while that of canine transmigration was 0.12-0.98 percent [7].

Gruszka et al. described that this developmental anomaly is relatively rare [8]. Patients with canine transmigration are often characterized also by lower lateral incisor hypodontia or lower second premolar hypodontia, enamel developmental defects, reduced teeth, and impacted upper canines. The intraosseous migration of a tooth apparently starts during the early mixed dentition stage and may take place over a period of many years [4]. The possible etiologic factors include genetics, premature loss of the deciduous teeth, persistent deciduous teeth, incorrect position of the dental lamina, hyperdontia, crowding, spacing in the dental arches, odontoma and alveolar crest trauma [8]. The mean age at which transmigration occurs is non-specific, ranging from adolescence to adulthood. Females appear to be affected more frequently than males [7].

In the present study, we report two cases of an asymptomatic transmigrated and impacted mandibular permanent canine. Both were managed by surgical extraction under local anesthesia. The incidence, etiology, and treatment of transmigrated and impacted canines are also discussed.

## Case presentation

Case 1

**Figure 1:**
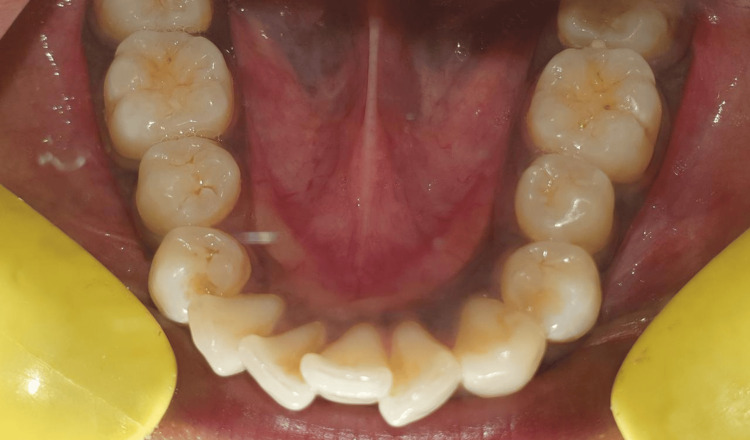
Preoperative image showing mandibular right canine missing.

A 22-year-old woman reported to the department complaining of protruded teeth. On clinical examination, her mandibular right permanent canine was found to be missing. Mandibular right incisors were crowded and positioned out of alignment with the rest of the teeth (Figure 1). 

**Figure 2:**
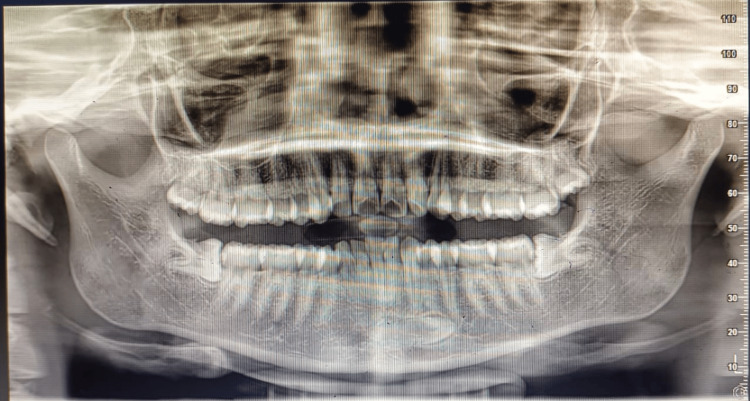
OPG showing impacted and transmigrated canine. OPG: Orthopantomogram.

Such positioning of incisors caused confusion in identifying the teeth. Panoramic radiography indicated that the right mandibular permanent canine was impacted and had migrated between the root apices of the contralateral lateral incisor and second premolar (Figure 2). 

**Figure 3:**
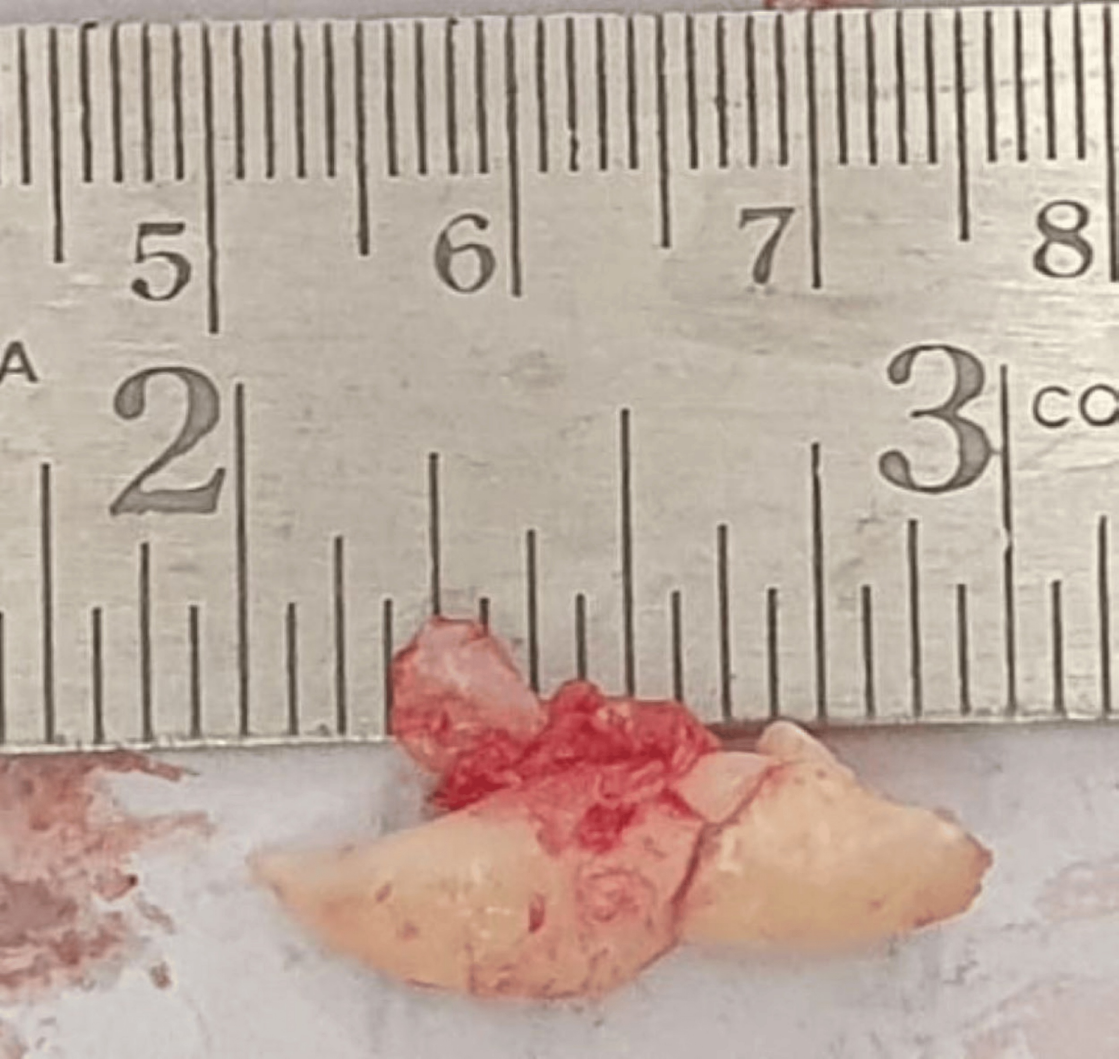
Surgically extracted tooth.

The patient was asymptomatic. Following consultation with the orthodontist, the transmigrated canine was surgically extracted under local anesthesia. Bilateral inferior alveolar and lingual nerve blocks were administered to facilitate the sectioning of the impacted canine (Figure 3). The wound was closed with a 3-0 polyglactin suture. 

Case 2

**Figure 4:**
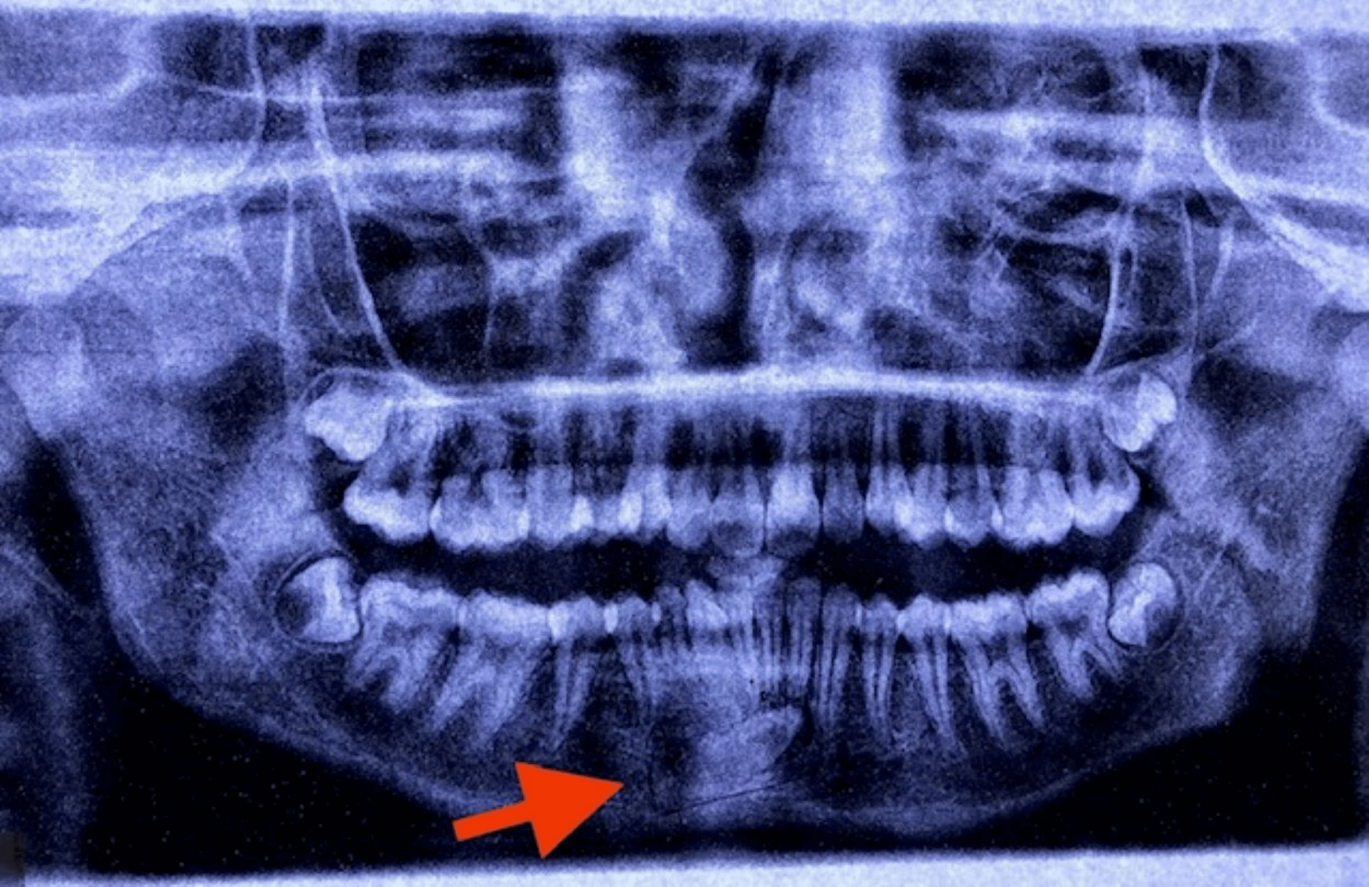
OPG showing impacted and transmigrated canine. OPG: Orthopantomogram.

A 17-year-old woman presented to the department complaining of protruded teeth. On clinical examination, her right mandibular permanent canine was missing, and the right mandibular deciduous canine was retained. Panoramic radiography indicated that the right mandibular permanent canine was impacted and had migrated between the root apices of the left central and lateral incisors (Figure 4).

**Figure 5:**
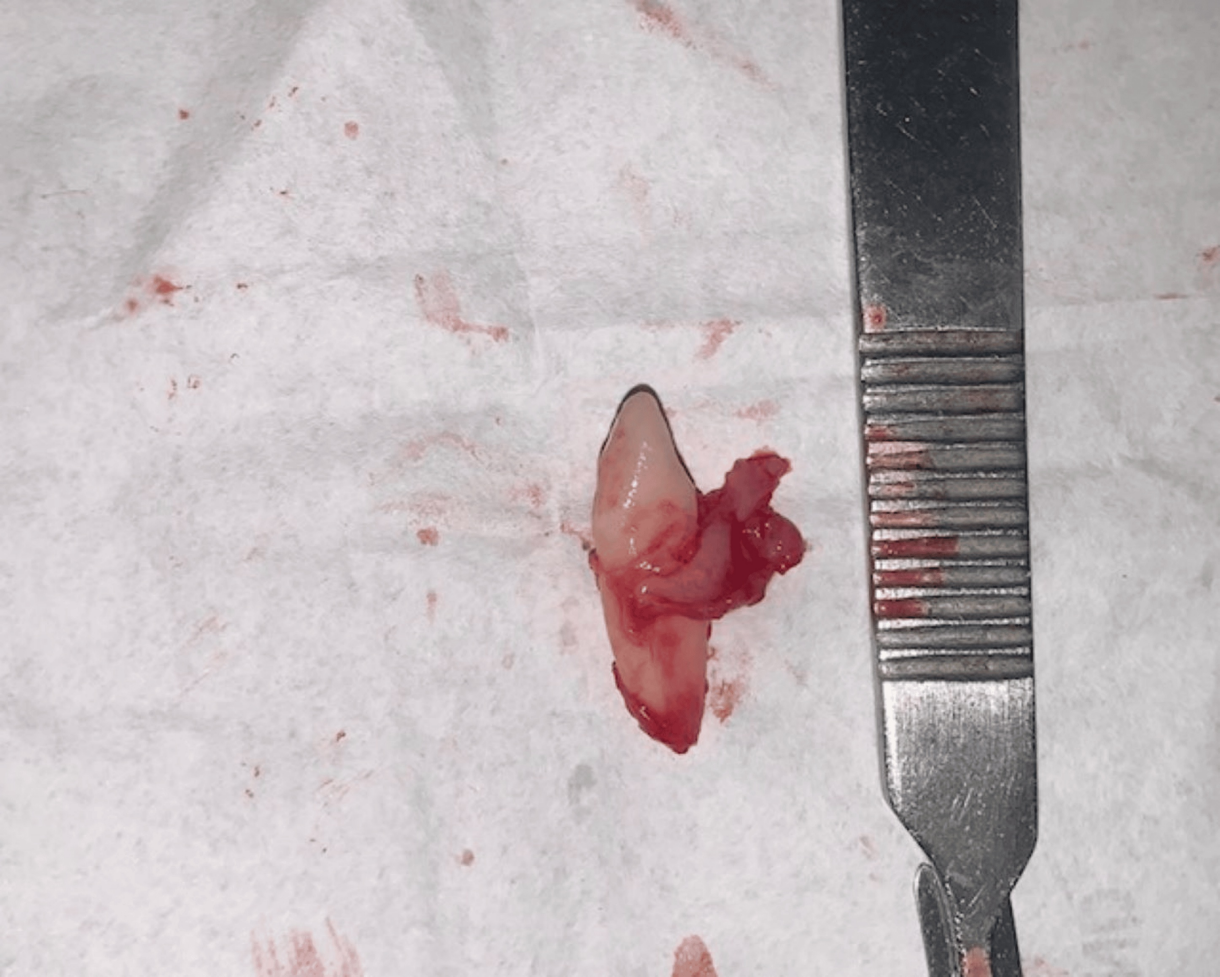
Surgically extracted tooth.

The patient was asymptomatic. Following consultation with the orthodontist, the transmigrated canine was surgically extracted under local anesthesia. Bilateral inferior alveolar and lingual nerve blocks were administered to facilitate the extraction (Figure 5). The wound was closed with a 3-0 polyglactin suture. 

## Discussion

The occurrence of impacted mandibular canine is rarer than that of maxillary canine. It is an even rarer phenomenon when such an impacted mandibular canine migrates to the other side of the mandible, crossing the mandibular midline [9]. According to Ayadin et al., the mandibular canine is the tooth most commonly documented to migrate and cross the midline [5]. He further explained that the larger cross-sectional area of the anterior mandible compared with the anterior maxilla may be the reason for the higher frequency of mandibular canine transmigration. Costello et al. described that transmigration of maxillary canine is uncommon due to the shorter distance between the roots of maxillary incisors and the floor of the nasal fossa and restriction of the path of tooth movement by the roots of adjacent teeth, the maxillary sinus and mid-palatine suture, which acts as a barrier [10].

In 2002, Mupparapu classified lower canine transmigration based on the analysis of 127 clinical cases [11]. According to his classification, there are five types of transmigrated canines, which are presented below.

Type 1- Mesially inclined impacted canine lying lingual or buccal to the lower anterior teeth; a certain part of the canine crown crosses the midline.

Type 2- The impacted canine lies below the apices of lower incisors close to the base of the mandible.

Type 3- The canine erupted mesially or distally when compared to the canine on the opposite side.

Type 4- The canine is positioned horizontally below lower premolars or molars on the opposite side, close to the base of the mandible.

Type 5- The canine is positioned vertically in the midline.

In the present study, the radiographic appearance of the transmigrated canine in Case 1 and Case 2 were in accordance with Type 4 and Type 2, respectively.

Gruszka et al. said that analyzing the degree of canine inclination on a panoramic radiograph is useful in the early diagnosis of transposition [8]. He further explained that an angle of inclination between 25 and 30 degrees to midline should not cause transmigration. The angle of canine inclination between 30 and 50 degrees may indicate the presence of transmigration. When the inclination angle is larger than 50 degrees, the presence of transmigration is certain. The pathogenesis of transmigration is unclear. The most commonly accepted explanation is the abnormal displacement of dental lamina in embryonic life and the non-eruption of such canines [4]. Nodine emphasized that even a very small obstacle, such as a small root fragment or an odontoma, would be sufficient to divert a tooth from its normal path of eruption [12]. Javid [6] and Joshi [9] suggested that the cause of transmigration may be an abnormally strong eruption force, which drives the canine through the dense symphysis. They also noted that the conical shape of the crown and root of the mandibular canine facilitates its transmigration process within the mandible.

Clinical findings, such as the absence of the permanent mandibular canine, retention of the deciduous canine, and sometimes, congenitally missing lateral incisors [2,6,13] and mandibular premolars [14] have been reported in cases of mandibular transmigration. Javid observed that most transmigrated canines are asymptomatic, although follicular cyst formation and chronic infection with fistulisation have been reported [6].

Diagnosis is made based on clinical findings, panoramic radiographs, and CBCT. Umashree et al. suggested that transmigration must be suspected in cases where the mandibular canine is clinically absent from the dental arch, along with an abnormal retention of the mandibular deciduous canine [4]. She further emphasized the importance of a panoramic radiograph [OPG] in the diagnosis of canine transmigration, as a routine periapical radiograph fails to reveal transmigrated canines. Sathyanarayana et al. observed that CBCT images are helpful in accurately diagnosing and treatment planning [7]. In the present study, a permanent canine is missing in Case 1, and the space is occupied by a lateral incisor, which is abnormally positioned to simulate the canine. There was abnormal retention of the deciduous canine in Case 2. The treatment options for impacted and transmigrant mandibular canines include active observation, transplantation, surgical exposure and orthodontic alignment, and surgical extraction [3]. Selecting the optimal treatment requires a thorough clinical and radiographic assessment, often involving a multidisciplinary team (e.g., oral surgeons, orthodontists, and prosthodontists). Factors such as the patient’s age, the canine’s position, esthetic considerations, and patient preference all play significant roles in determining the most suitable treatment option.

Observation

Some authors have advocated that if the non-erupted tooth is symptomless and not associated with any pathology, it can be left in place. In these patients, a series of successive radiographs should be taken periodically [3,4,15]. A progressive worsening of the position of the unerupted canine or suggestion of a cystic change of the follicle should lead the clinician to consider the possibility of extraction [3].

Transplantation

Camilleri et al. described that transplantation may be undertaken when mandibular incisors are in normal position and space for the transmigrated canine is sufficient [3]. Umashree et al. suggested that the timing of a transplant is crucial because one main goal is to achieve maximum root length [4]. Ideally, the procedure should be done when the tooth’s root is between half and three-quarters complete, as this allows better chances for blood supply to re-establish. As the root apex gets closer to full closure, the prognosis for a successful transplant declines.

Surgical exposure and orthodontic alignment

Orthodontic treatment with surgical exposure and alignment is suitable for younger patients with minimal transmigration and a favorable position of canine within the jaw. Wertz described that transmigrated canines can be brought to their expected positions [16]. However, if the tip of the crown has migrated past the opposite incisor or if the apex of the migrated tooth is located past the apex of the adjacent lateral incisor, it might be mechanically impossible to return the transmigrated tooth to its expected position. Dalessandri et al. explained that since canine impaction is often identified when the root has already developed beyond two-thirds of its length, this advanced development can pose challenges for orthodontic traction and repositioning. Orthodontic traction is more difficult and complicated, although it is the most efficient strategy to restore physical occlusion [1].

Surgical extraction

Surgical extraction appears to be the most favored treatment for these teeth. Manne et al. suggested that the surgical extraction of transmigrated and impacted canine is indicated if the tooth is ankylosed, if the root is severely dilacerated if the impacted canine is lodged between roots of central and lateral incisors and if the orthodontic movement will jeopardize these teeth, or pathologic changes (such as cystic formation and infection), is observed [17]. Yavuz et al. reported that among the 71 impacted mandibular canines in their study, extraction was the most common treatment 57.7%), followed by surgical exposure and orthodontic traction (32.4%), observation (8.4%), and autotransplantation (1.4%) [18]. Caldwell et al. [19] and Bruszt et al. [20] showed that the transmigrated canine maintains its nerve supply from the original site. Hence, it is necessary to anesthetize the nerve on the side to which the canine belongs. In both cases, the canines had migrated horizontally to the position of the opposite second premolar and lateral incisor, respectively. As a result, orthodontic repositioning was not feasible, and surgical extraction was performed.

## Conclusions

The canines are important for aesthetics and function. An altered eruption position of these teeth is an important patient concern. Transmigration of the mandibular canine across the mandibular midline is rare. Most of the time, transmigrated canines are symptomless and detected as incidental findings, e.g., when the patient visits for orthodontic treatment. Early detection and timely extraction of the transmigrated impacted canine may prevent pathological changes, such as cysts and odontoma. Future studies are needed for a better understanding of this anomaly and to improve diagnosis and treatment approaches.
